# ER-Targeted PET for Initial Staging and Suspected Recurrence in ER-Positive Breast Cancer

**DOI:** 10.1001/jamanetworkopen.2024.23435

**Published:** 2024-07-26

**Authors:** Gary A. Ulaner, Mel Silverstein, Chaitali Nangia, Merry Tetef, Louis Vandermolen, Colleen Coleman, Sadia Khan, Heather MacDonald, Trushar Patel, Tust Techasith, Audrey Mauguen

**Affiliations:** 1 Molecular Imaging and Therapy, Hoag Family Cancer Institute, Irvine, California; 2 Radiology and Translational Genomics, University of Southern California, Los Angeles; 3 Surgery, Hoag Family Cancer Institute, Newport Beach, California; 4 Medicine, Hoag Family Cancer Institute, Newport Beach, California; 5 Department of Medicine, University of California, Los Angeles; 6 Department of Medicine, University of Southern California, Los Angeles; 7 Radiology, Hoag Family Cancer Institute, Newport Beach, California; 8 Department of Epidemiology and Biostatistics, Memorial Sloan Kettering Cancer Center, New York, New York

## Abstract

**Importance:**

There are insufficient data comparing 16α-^18^F-fluoro-17β-estradiol (FES) positron emission tomography (PET) computed tomography (CT) with standard-of-care imaging (SOC) for staging locally advanced breast cancer (LABC) or evaluating suspected recurrence.

**Objective:**

To determine the detection rate of FES PET/CT and SOC for distant metastases in patients with estrogen receptor (ER)-positive LABC and recurrences in patients with ER-positive BC and suspected recurrence.

**Design, Setting, and Participants:**

This diagnostic study was conducted as a single-center phase 2 trial, from January 2021 to September 2023. The study design provided 80% power to find a 20% detection rate difference. Participants included patients with ER-positive LABC (cohort 1) or suspected recurrence (cohort 2). Data were analyzed from September 2023 to February 2024.

**Exposure:**

Participants underwent both SOC imaging and experimental FES PET/CT. When there were suspicious lesions on imaging, 1 was biopsied for histopathological reference standard to confirm presence (true positive) or absence (false positive) of malignant neoplasm.

**Main Outcomes and Measures:**

The outcome of interest was the detection rate of FES PET CT vs SOC for distant metastases and recurrences.

**Results:**

A total of 124 patients were accrued, with 62 in cohort 1 (median [IQR] age, 52 [32-84] years) and 62 in cohort 2 (median [IQR] age, 66 [30-93] years). In cohort 1, of 14 true-positive findings, SOC imaging detected 12 and FES detected 11 (*P* > .99). In cohort 2, of 23 true-positive findings, SOC detected 16 and FES detected 18 (*P* = .77). In 30 patients with lobular histology, of 11 true-positive findings, SOC detected 5 and FES detected 9 (*P* = .29). There were 6 false-positive findings on SOC and 1 false-positive finding on FES PET/CT (*P* = .13).

**Conclusions and Relevance:**

In this diagnostic study with pathological findings as the reference standard, no difference was found between FES PET/CT and current SOC imaging for detecting distant metastases in patients with ER-positive LABC or recurrences in patients with ER-positive tumors and suspected recurrence. FES PET/CT could be considered for both clinical indications, which are not part of current Appropriate Use Criteria for FES PET. The findings regarding FES PET/CT in patients with lobular tumors, and for lower false positives than current SOC imaging, warrant further investigation.

## Key Points

**Question:**

Does estrogen receptor (ER)–targeted 16α-^18^F-fluoro-17β-estradiol (FES) positron emission tomography (PET) computed tomography (CT) perform as well as current standard-of-care imaging (SOC) for staging locally advanced breast cancer or evaluating suspected recurrence?

**Findings:**

In this diagnostic study of 124 patients with ER-positive breast cancer, there was no statistically significant difference between SOC and experimental FES PET/CT for detection of pathologically proven distant metastases or recurrences.

**Meaning:**

These findings suggest that FES PET/CT could be considered for staging or detection of recurrence in patients with ER-positive breast cancer.

## Teaser

This diagnostic study assesses the detection rates for distant metastases in patients with estrogen receptor (ER)–positive breast cancer and recurrences in patients with ER-positive breast cancer using 16α-^18^F-fluoro-17β-estradiol positron emission tomography (PET) compared with standard-of-care imaging.

## Introduction

Systemic staging of patients with breast cancer (BC) plays an essential role for patients with newly diagnosed locally advanced BC (LABC) and BC with suspected recurrences.^1,2,3,4,5^ As patients with LABC have a high risk for distant metastases at initial diagnosis, they undergo systemic staging with imaging.^6^ If imaging confirms local disease only, curative-intent therapy with systemic endocrine or chemotherapy, surgery, and radiation therapy is performed.^7^ If imaging confirms distant metastases, less aggressive palliative systemic therapies are used.^8^ For patients with suspected recurrence, imaging is used to confirm recurrence, as well as to determine the location and extent of detected disease. These findings impact whether patients will undergo therapy and what therapy modalities are chosen.^6^

For patients with either LABC or suspected recurrence of BC, National Comprehensive Cancer Network (NCCN) guidelines suggest either computed tomography (CT) of the chest, abdomen, and pelvis with nuclear bone scan or ^18^F-fluorodeoxyglucose (FDG) positron emission tomography (PET) as imaging modalities for systemic staging of disease location and extent.^6^ There are studies suggesting FDG PET/CT may be superior to CT bone scan^3,9,10^; however, NCCN guidelines continue to first emphasize CT bone scan, with FDG PET/CT described as “useful in certain circumstances.”^6^ Given NCCN recommendations, many insurances still require CT bone scan to be used for these clinical indications. More recently, imaging targeted by estrogen receptor (ER) status has become clinically available with US Food and Drug Administration (FDA) approval of 16α-^18^F-fluoro-17β-estradiol (FES) PET.^11,12^ As 80% of breast malignant neoplasms are ER-positive, FES PET may be valuable in multiple clinical scenarios.^11,12^ Appropriate Use Criteria for FES PET/CT, developed by the Society of Nuclear Medicine and Molecular Imaging,^13^ concluded this imaging modality is useful for evaluation of lesions inconclusive on other tests, for evaluation of lesions that are difficult or dangerous to biopsy, and as a prognostic biomarker when endocrine therapy is considered in patients with ER-positive metastatic breast cancer.^13^ There are also preliminary studies suggesting FES PET/CT may be superior to FDG PET/CT in patients with invasive lobular carcinoma (ILC) of the breast.^14,15^ To our knowledge, there are no data comparing FES PET with current standard-of-care imaging (SOC), such as CT bone scan or FDG PET/CT, for staging LABC or evaluating suspected recurrences. This study was designed to determine and compare the detection rate of FES PET/CT vs SOC imaging for distant metastases in patients with ER-positive LABC and recurrences in patients with ER-positive BC and suspected recurrence, using histopathological analysis as the reference standard for confirming a malignant neoplasm.

## Methods

This diagnostic study was performed after approval by the WCG institutional review board. All participants provided written informed consent. This study is reported following the Standards for Reporting of Diagnostic Accuracy (STARD) reporting guideline.

### Participants

This diagnostic study was conduced as a single-center phase 2 trial (ClinicalTrials.gov identifier: NCT04883814), performed at the Hoag Family Cancer Institute in Irvine, California. Patients were referred to Hoag Molecular Imaging and Therapy by medical and surgical oncologists. Hoag Molecular Imaging and Therapy then screened patients for eligibility, obtained written informed consent, and coordinated trial components. Inclusion criteria were women aged 18 years or older with biopsy-proven ER-positive LABC (cohort 1) or with BC and suspected recurrent disease due to symptom, abnormal tumor marker, or equivocal imaging finding (cohort 2). LABC was defined as anatomic stage IIB to IIIC by the American Joint Committee on Cancer.^16^ Exclusion criteria were pregnancy or lactation, unwillingness to provide written informed consent, men, and current use of tamoxifen or fulvestrant, which bind estrogen and may prevent accurate FES PET scanning.^17^ Patients with all histological types of BC were allowed to enroll. Baseline characteristics recorded included age at enrollment, ER immunohistochemistry (IHC; range, 1+ to 3+), progesterone receptor IHC (range, 1+ to 3+), ERBB2 (formerly known as *HER2*) IHC (range, 0 to 3+), ERBB2 status by ASCO Guidelines^18^ (negative or positive), and histology (no special type [previously referred to as *invasive ductal carcinoma*], ILC, mixed, and mucinous); for cohort 1, we also included the pretrial anatomic stage (IIB, IIIA, IIIB, or IIIC).

### Imaging and Interpretation

All enrolled participants underwent SOC imaging (either CT bone scan or FDG PET/CT, as selected by the referring physician) and an experimental FES PET/CT within 14 days of each other with no intervening therapy. For participants receiving CT bone scan, both CT with intravenous contrast of the chest, abdomen, and pelvis, as well as ^99m^Tc methylene diphosphate nuclear bone scan were performed by standard protocols. For participants receiving FDG PET/CT, patients fasted for at least 4 hours prior to administration of 10 millicurie (mci) (±10%) of FDG, followed by a 60-minute tracer uptake period, then CT and PET imaging from upper thigh proceeding to the skull base on a Siemens Biograph mCT Flow PET/CT system. All participants underwent FES PET/CT, including administration of 5 mci (±10%) of FES, followed by a 60-minute tracer uptake period, then CT and PET imaging on a Siemens Biograph mCT Flow PET/CT system, as described in the Society of Nuclear Medicine and Molecular Imaging Procedure Standard for FES PET.^19^ Both FDG PET/CT and FES PET/CT were performed without the administration of intravenous contrast. Physicians board certified in diagnostic radiology and nuclear medicine separately interpreted SOC imaging and FES PET/CT (G.A.U.), with blinding to the other modality but not to clinical information. If lesions suspicious for distant metastases (cohort 1) or recurrent malignant neoplasm (cohort 2) were identified on either SOC imaging or FES PET/CT, then 1 suspicious lesion was selected for CT-guided biopsy. Biopsy was performed by a board-certified interventional radiologist (T.P. and T.T.) using imaging to guide biopsy. Histopathology from biopsy samples was used as the reference standard to confirm presence (true positive) or absence (false positive) of a malignant neoplasm.

### Statistical Analysis

The primary end points were the presence of a lesion on FES PET/CT and on SOC imaging histologically proven to represent distant metastasis (cohort 1) or recurrence (cohort 2). For each imaging modality, the true-positive rate was defined as the proportion of patients with biopsy-confirmed disease who had at least 1 lesion detected on imaging. Those detection rates were estimated with exact 95% CIs and were compared between FES PET/CT and SOC imaging using McNemar tests. Positive predictive values were calculated for FES PET/CT and SOC imaging. The cohort sizes of 62 patients in each cohort of the study were selected to allow 80% power to detect a 20% difference in detection rate between SOC imaging and FES PET/CT while controlling the 1-sided type I error rate at 5%. Secondary end points were the sites of distant metastases or recurrences detected by FES PET/CT that were undetected by CT bone scan and incidence of adverse events from FES administration and imaging. Those are reported using descriptive statistics.

Analyses were conducted using R software version 4.2.0 (R Project for Statistical Computing). Data were analyzed from September 2023 to February 2024.

## Results

**Figure 1.**
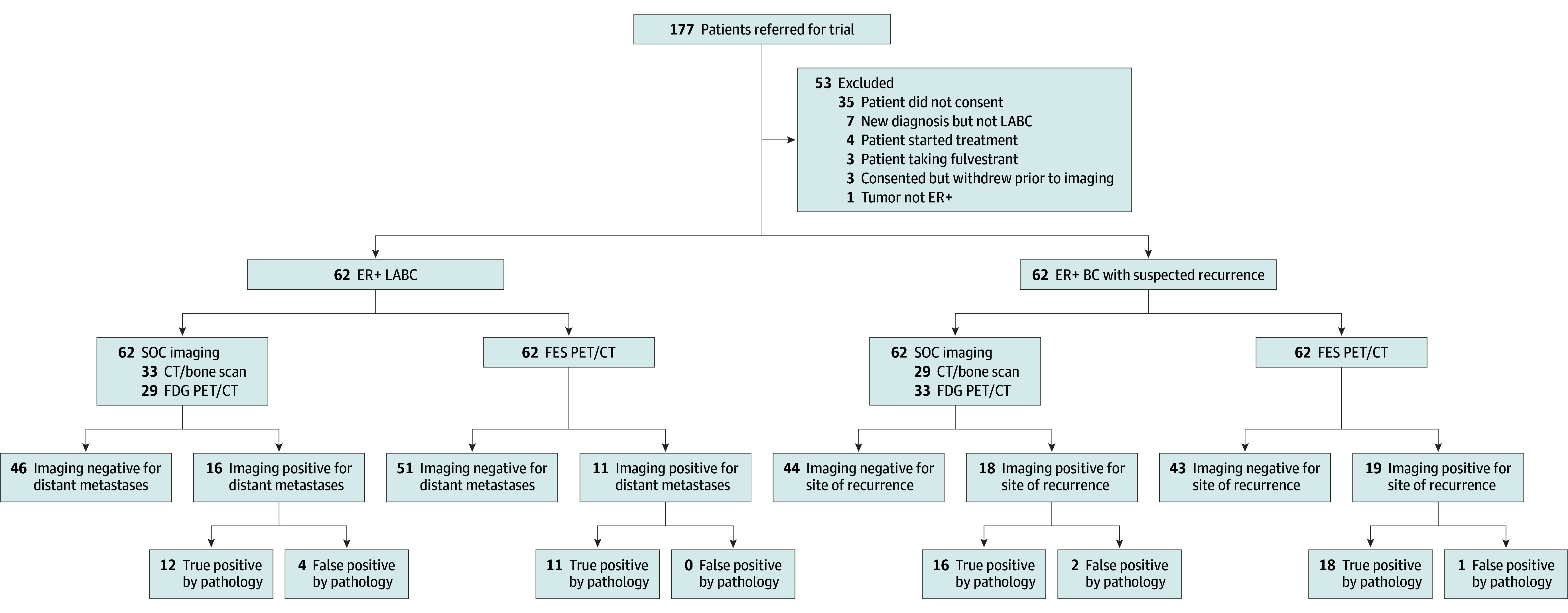
Participant Recruitment Flowchart BC indicates breast cancer; CT, computed tomograph; ER+, estrogen receptor positive; FDG, ^18^F-fluorodeoxyglucose; FES, 16α-^18^F-fluoro-17β-estradiol; LABC, locally advanced BC; PET, positron emission tomography; SOC, standard of care.

A total of 177 patients were referred for consideration of eligibility for this study between January 13, 2021, and September 1, 2023. A total of 53 patients were excluded, and 124 patients were included in the analysis (Figure 1). This included 62 participants evaluated in cohort 1 (median [IQR] age, 52 [32-84] years) and 62 participants evaluated in cohort 2 (median [IQR] age, 66 [30-93] years). Baseline characteristics for both cohorts are detailed in the Table. All but 3 patients demonstrated ER IHCs of 2+ or 3+. BC histological findings were no special type (92 participants), ILC (30 participants), and other (2 participants). Biopsy was performed in all patients with positive imaging findings.

**Table.** Baseline Participant Characteristics

Characteristic	Participants, No. (%)	
	Cohort 1 (n = 62)	Cohort 2 (n = 62)
ER		
1	0	3 (4.8)
2	23 (37.1)	19 (30.7)
3	39 (62.9)	40 (64.5)
PR		
0	5 (8.1)	14 (22.6)
1	9 (14.5)	5 (8.1)
2	12 (19.3)	13 (21.0)
3	36 (58.1)	30 (48.4)
ERBB2 IHC		
0	31 (50.0)	28 (45.2)
1	20 (32.2)	25 (40.3)
2	6 (9.7)	7 (11.3)
3	5 (8.1)	2 (3.2)
ERBB2 status		
Negative	54 (87.1)	56 (90.3)
Positive	8 (12.9)	6 (9.7)
Histology		
NST	49 (79.0)	43 (69.4)
ILC	13 (21.0)	17 (27.4)
Mixed	0	1 (1.6)
Mucinous	0	1 (1.6)
Histologic grade		
1	6 (9.7)	13 (21.0)
2	40 (64.5)	31 (50.0)
3	16 (25.8)	8 (12.9)
Unknown	0	10 (16.1)
Anatomic tumor stage		
IIB	33 (53.2)	NA
IIIA	23 (37.1)	NA
IIIB	6 (9.7)	NA
Age at FES, median (range), y	52 (32-84)	66 (30-93)

Abbreviations: ER, estrogen receptor; ERBB2, human epidermal growth factor 2; FES, 16α-^18^F-fluoro-17β-estradiol imaging; IHC, immunohistochemistry; ILC, invasive lobular carcinoma; NA, not applicable; NST, no special type (previously referred to as *invasive ductal carcinoma*); PR, progesterone receptor.

In cohort 1, SOC imaging was CT bone scan in 33 participants and FDG PET/CT in 29 participants. SOC imaging found that 46 participants (74.2%) were negative for distant metastases and 16 participants (25.8%) had lesions suspected to be distant metastases. Based on pathology of biopsy lesions, 12 lesions (75.0%) were true positives for distant metastases, while 4 lesions (25.0%) were false positives. FES PET/CT found that 51 participants (82.3%) were negative for distant metastases and 11 participants (17.7%) were positive for lesions suspected to be distant metastases. On pathological examination, all 11 lesions were true positives for distant metastases. There were no false positives on FES PET/CT in cohort 1. Overall, 14 of 62 patients (22.6%) had pathologically proven distant metastases, with 9 detected by both modalities, 3 detected only by SOC imaging, and 2 detected only by FES PET/CT. For the primary end point of detection rate for distant metastases, SOC imaging detected 12 of 14 patients (85.7%; 95% CI, 57.2%-98.2%), while FES PET/CT detected 11 of 14 patients (78.5%; 95% CI, 49.2%-95.3%). There was no statistically significant difference between SOC imaging and FES PET/CT (McNemar test *P* > .99).

In cohort 2 (suspected recurrence), 29 participants underwent as SOC imaging a CT bone scan and 33 FDG PET/CT. SOC imaging showed that 44 participants (71.0%) were negative for recurrence, while 18 participants (29.0%) had lesions suspected to be recurrence. Based on pathological findings in biopsy lesions, 16 lesions (88.9%) were true-positive tumor recurrences, while 2 lesions (11.1%) false-positive recurrences. FES PET/CT imaging demonstrated that 43 participants (69.4%) were negative for recurrence, while 19 participants (30.6%) had lesions suspected to be recurrence. Pathological examinations found 18 lesions (94.7%) were true-positive tumors in the biopsy and 1 lesion (5.3%) was a false positive. Overall, 23 patients (37.1%) had a pathologically proven recurrent malignant neoplasms, with 11 detected by both modalities, 5 detected only by SOC imaging, and 7 detected only by FES PET/CT. For the primary end point of detection rate for recurrent malignant neoplasm, SOC imaging detected 16 recurrences (69.6%; 95% CI, 47.X%-87.X%), while FES PET CT detected 18 recurrences (78.2%; 95% CI, 56.X%-93.X%). There was no statistically significant difference between SOC imaging and FES PET CT (McNemar test *P* = .77).

**Figure 2.**
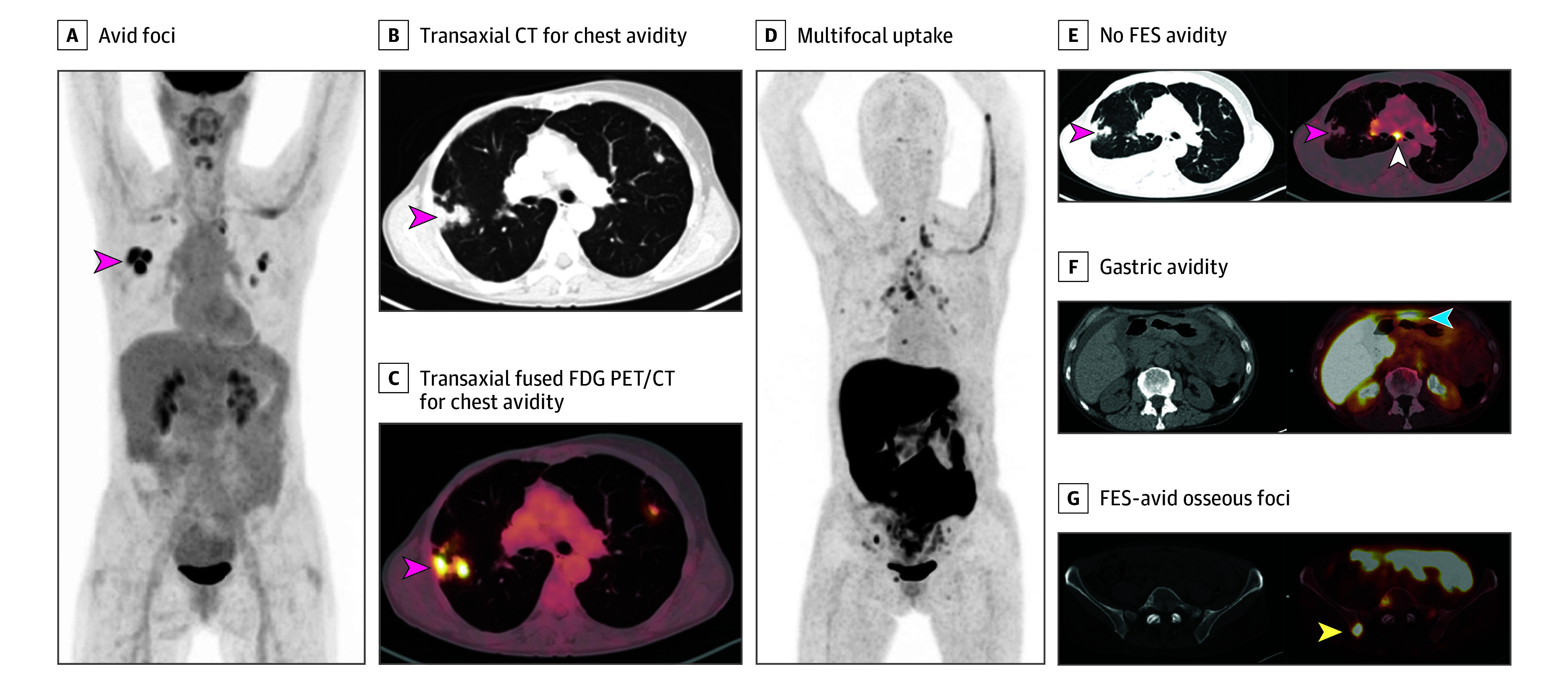
Recurrent Disease Detected on 16α-^18^F-fluoro-17β-estradiol (FES) Position Emission Tomograph (PET) Computed Tomography (CT) but Not Detected on ^18^F-fluorodeoxyglucose (FDG) PET/CT Patient was a woman in her 50s with suspected recurrence of invasive lobular breast cancer. A, Maximum intensity projection image from FDG PET/CT demonstrating avid foci in the chest (red arrow). Transaxial CT (B) and transaxial fused FDG PET/CT image (C) showing that the chest avidity corresponds to FDG-avid lung nodules (red arrows) suspicious for malignancy. The lung nodule was subsequently biopsied but found to represent benign granulomatous inflammation and thus a false positive on FDG PET/CT. D, Maximum intensity projection image from FES PET/CT demonstrating multifocal uptake suspicious for malignancy. E, Transaxial CT and fused FES PET/CT demonstrating no FES-avidity in the biopsy proven benign granulomatous lung nodules (red arrows), thus true negative on FES PET/CT. FES-avidity in nodes (white arrow) is suspicious for malignancy. F, Transaxial CT and fused FES PET/CT demonstrates gastric avidity (blue arrow) suspicious for malignancy. G, Transaxial CT and fused FES PET/CT demonstrates FES-avid osseous foci (yellow arrow) suspicious for malignancy. This osseous focus was subsequently biopsied and proved to be an osseous metastasis and thus true positive on FES PET/CT.

There were 13 participants in cohort 1 and 17 participants in cohort 2 with ILC histology (Table). In subgroup analysis of patients with ILC from both cohorts, 11 patients (36.6%) had pathologically proven distant metastases or recurrent malignant neoplasm, with 3 patients detected by both modalities, 2 patients detected only by SOC imaging, and 6 patients detected only by FES PET/CT, including the one illustrated in Figure 2. For the end point of detection rate for distant metastases (cohort 1) and recurrent malignant neoplasm (cohort 2), SOC imaging detected 5 patients (45.4%), while FES PET/CT detected 9 patients (81.8%). There was not enough evidence to conclude that FES PET CT was better than SOC imaging (McNemar test *P* = .29).

With pathological findings as the reference standard, SOC imaging had 4 false positives in cohort 1 and 2 false positives in cohort 2, such as the example in Figure 2, while FES PET/CT had no false positives in cohort 1 and 1 false positive in cohort 2. When both cohorts were combined, the McNemar *P* value for a difference between false-positive rates from SOC imaging (6 false positives) and FES PET/CT (1 false positive) was *P* = .13. When both cohorts were combined, the positive predictive value of FES PET/CT was 96.7% (29 of 30 patients) and the positive predictive value of SOC imaging was 82.4% (28 or 34 patients).

Among 62 patients in cohort 1, 6 (9.7%) had extra-axillary nodes detected on FES PET/CT that were not detected on SOC imaging. As detection of extra-axillary nodes was not a primary aim of the trial, these were not biopsied for pathologic confirmation. There were no participants with extra-axillary nodes detected on SOC imaging but not FES PET/CT.

**Figure 3.**
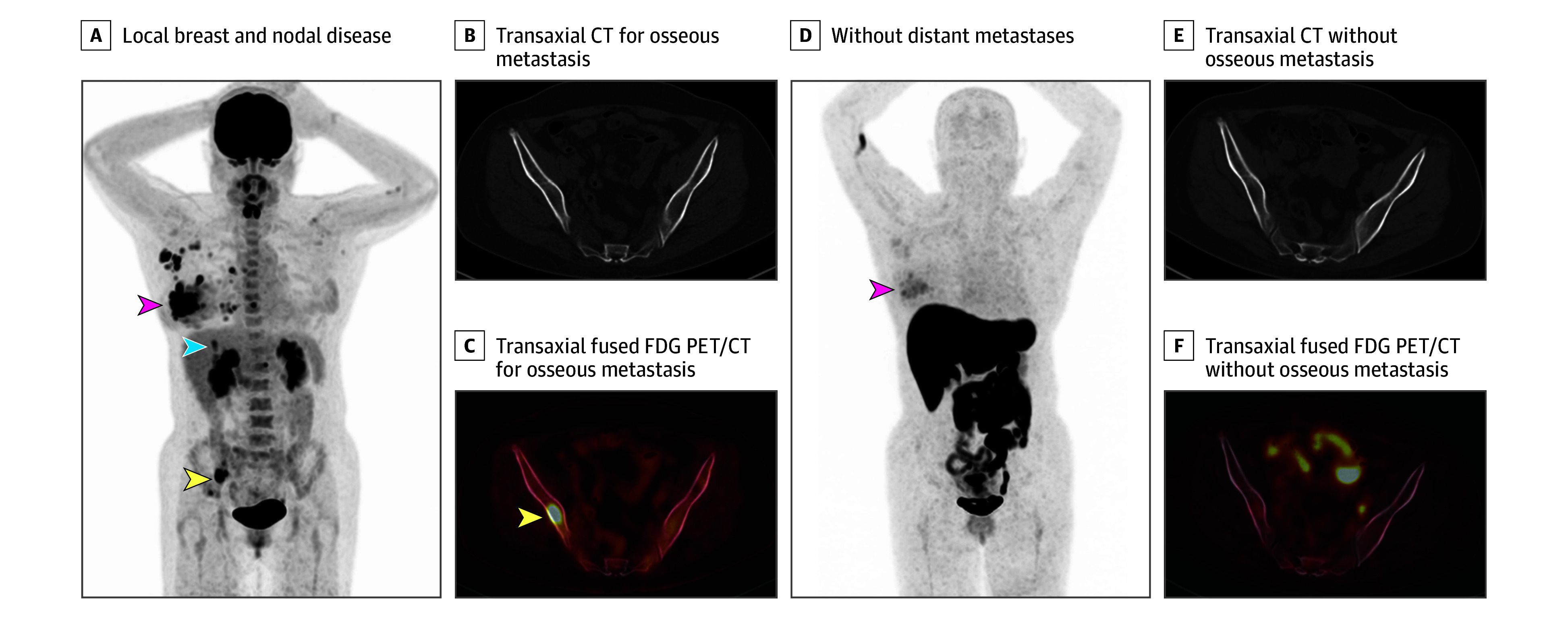
Distant Metastases Detected on 16α-^18^F-fluoro-17β-estradiol (FES) Position Emission Tomograph (PET) Computed Tomography (CT), but Not Detected on ^18^F-fluorodeoxyglucose (FDG) PET/CT Patient was a woman in her 50s with newly diagnosed, locally advanced breast cancer and no special type histology. A, Maximum intensity projection image from FDG PET/CT demonstrating local breast and nodal disease (red arrow), as well as foci suspicious for hepatic (blue arrow) and osseous (yellow arrow) metastases. Transaxial CT (B) and transaxial fused FDG PET/CT (C) through the pelvis demonstrating an osseous metastasis (yellow arrow), which was subsequently proven with biopsy to be malignant. D, Maximum intensity projection image from FES PET/CT demonstrating local breast and nodal disease (red arrow) without evidence of distant metastases. Transaxial CT (E) and transaxial fused FES PET/CT (F) through the pelvis are without evidence of the biopsied FDG-avid osseous metastasis.

For the 9 participants in whom distant metastases or recurrences were detected by FES PET/CT but undetected by SOC, the sites of distant metastases were bone (4 patients); bone and nodes (3 patients); bone, pleura, peritoneum, adrenal, and gastrointestinal tract (1 patient) (Figure 2); and chest wall, subcutaneous tissue, and lung (1 patient). For these 9 participants, SOC imaging was CT bone scan in 5 participants and FDG PET/CT in 4 participants. For the 8 participants in whom distant metastases or recurrences were detected by SOC but undetected by FES PET/CT, the sites of metastases were nodes (3 patients), bone (2 patients), bone and liver (1 patient) (Figure 3), bone and adrenal (1 patient), and lung (1 patient).

There were no adverse events reported from FES administration or FES PET CT imaging. There were no adverse events reported from any SOC imaging.

## Discussion

This diagnostic study using pathological findings as the reference standard found no significant difference between FES PET/CT and current SOC imaging methods for the detection rate of distant metastases in patients with LABC or recurrences in patients with suspected recurrence. The Institute of Medicine^20^ and the American Society of Clinical Oncology^21^ have emphasized comparative effectiveness research for PET/CT. This study provides comparative effectiveness research for FES PET/CT and suggests FES PET/CT may be considered in patients with ER-positive BC for both systemic staging at initial diagnosis of LABC and evaluation of suspected recurrences.

ER-targeted imaging with FES PET/CT is a recently FDA-approved modality, with NCCN guidelines^6^ and Appropriate Use Criteria^13^ suggesting several appropriate clinical uses, such as assessing lesions that are difficult to biopsy or evaluation of lesions equivocal on other tests. However, to our knowledge, there were previously no data comparing FES PET/CT with current SOC imaging modalities for staging LABC or evaluating suspected recurrences. This study provides evidence that FES PET/CT performs comparably with current SOC imaging methods for both of these clinical indications.

FES detects ER available to bind estrogen ligand, thus FES PET/CT is indicated only for patients with known ER-positive BC.^12^ Patients with ER-negative BC are unlikely to have their disease detected by FES PET, were excluded from this study, and should not be considered for FES PET/CT.^17^

Dayes et al^9^ conducted a prospective trial comparing FDG PET/CT vs CT bone scan for systemic staging of patients with LABC was conducted from 2016 to 2022 and found that FDG PET/CT detected more distant metastases than CT bone scan. A subgroup analysis in our study comparing SOC imaging by FDG PET/CT vs SOC imaging by CT bone scan was limited by the number of events recorded in the subgroups. Our trial began in 2021, before Dayes et al^9^ reported their findings and defined SOC imaging as either CT bone scan or FDG PET/CT, as both are recognized as SOC in NCCN guidelines.^6^

Of note, it was common for distant metastases to be detected by only 1 imaging modality. For example, in cohort 2, of 23 patients with pathologically proven recurrent malignant neoplasms, only 11 (47.8%) were identified by both imaging modalities, while 5 (21.7%) were detected only by SOC, and 7 (30.4%) were detected only by FES PET/CT. As more than half of recurrent malignant neoplasms were detected by only 1 imaging modality, the question of how extensive imaging staging should be arises. When there is strong suspicion of recurrence of a malignant neoplasm based on symptoms and laboratory data and one imaging modality finds negative results, should a second imaging modality be attempted? Further data will be needed to address this dilemma.

The most common sites of distant metastases and recurrences detected by FES PET/CT not detected by SOC imaging were in bone, followed by lymph nodes. Physiologic excretion of FES in the liver reduces sensitivity of detection of liver metastases by FES. In our study, there were no participants with liver-only metastases or recurrences.

In cohort 1, systemic staging of LABC showed suspicious extra-axillary nodes in 10% of patients with FES PET/CT that were not detected using SOC imaging. While not a primary aim of the trial, the detection of previously unidentified extra-axillary nodal metastases affects staging, prognosis, and treatment.^3,22^ The ability of FES PET CT to identify extra-axillary nodal metastases requires further evaluation.

We found fewer false-positive findings with FES PET CT (1 false-positive) compared with SOC imaging (6 false positives). Further study of this issue will be needed.

This study was not designed to collect data on management changes. When previously unknown distant metastases are identified in patients with LABC, management and treatment strategies change for most patients.^9^ Thus, it is highly likely that the identification of distant metastases and sites of recurrence by FES PET will have substantial impact on clinical treatment of patients.

The strengths of this trial include the prospective design and, perhaps most importantly, histologic proof of imaging findings. While imaging is standardly used in patients with cancer, trials with biopsy confirmation are better able to resolve true-positive results from false-positive imaging findings.^23^

### Limitations

This study has some limitations The weaknesses of this study include the single-center design and the fact that participants in cohort 1 were all stage IIB to IIIB, while the design also called for patients with stage IIIC disease to be included. The patients included in the study had a lower disease rate than assumed during the study design, affecting the power for the primary aims; subgroup analyses were not powered to show differences. Additionally, this diagnostic study was not a randomized trial. Each patient received both SOC imaging and FES PET CT; thus, each patient served as their own internal control for comparison of the different imaging modalities.

## Conclusions

In this diagnostic study with pathological examination as the reference standard, no significant difference was found between FES PET/CT and current SOC imaging for the detection of distant metastases in patients with ER-positive LABC or recurrences in patients with ER-positive BC and suspected recurrence. FES PET/CT could be considered for these clinical indications. FES PET/CT may be most useful in patients with ILC, for whom FDG avidity is lower.^14,15^ A future trial of only participants with ILC is warranted, which may replicate this study design but be performed in a multiinstitutional trial enrolling only patients with ILC histology.

## Notes

Supplement.
Data Sharing Statement
