# Error in FES Dose in Methods

**DOI:** 10.1001/jamanetworkopen.2024.45816

**Published:** 2024-10-25

In the Original Investigation titled “ER-Targeted PET for Initial Staging and Suspected Recurrence in ER-Positive Breast Cancer,”^1^ published July 26, 2024, the Methods incorrectly described patients as receiving 9 millicurie (mci) of 16α-^18^F-fluoro-17β-estradiol (FES). This should have been described as 5 mci. This article has been corrected.^1^
