# Supplementary material for: ER-Targeted PET for Initial Staging and Suspected Recurrence in ER-Positive Breast Cancer
Source: JAMA Netw Open. 2024 Jul 26;7(7):e2423435. doi: 10.1001/jamanetworkopen.2024.23435 (PMC11282447; doi:10.1001/jamanetworkopen.2024.23435)
Supplement: Supplement. — Data Sharing Statement [file jamanetwopen-e2423435-s001.pdf]

Data Sharing Statement

Ulaner. ER-Targeted PET for Initial Staging and Suspected Recurrence in ER-Positive Breast Cancer. *JAMA Netw Open*. Published July 26, 2024.
doi:10.1001/jamanetworkopen.2024.23435

Data
Data available: Yes
Data types: Deidentified participant data
How to access data: gary.ulaner@hoag.org
When available: With publication

Supporting Documents
Document types: None

Additional Information
Who can access the data: anyone requesting the data
Types of analyses: for any purpose
Mechanisms of data availability: with a signed data access agreement
